# Effects of Dietary Supplementation of Oregano Essential Oil to Sows on Oxidative Stress Status, Lactation Feed Intake of Sows, and Piglet Performance

**DOI:** 10.1155/2015/525218

**Published:** 2015-10-11

**Authors:** Chengquan Tan, Hongkui Wei, Haiqing Sun, Jiangtao Ao, Guang Long, Siwen Jiang, Jian Peng

**Affiliations:** ^1^Department of Animal Nutrition and Feed Science, College of Animal Science and Technology, Huazhong Agricultural University, Wuhan 430070, China; ^2^YangXiang Joint Stock Company, Guigang 53700, China; ^3^Key Laboratory of Swine Breeding and Genetics of the Agricultural Ministry, College of Animal Science and Technology, Huazhong Agricultural University, Wuhan 430070, China

## Abstract

Fifty-four multiparous large white sows were used to determine the effects of supplementing oregano essential oil (OEO) to the gestation and lactation diets on oxidative stress status, lactation feed intake, and their piglet performance. Two groups were fed diets with (OEO; *n* = 28) or without (Control; *n* = 26) supplemental 15 mg/kg OEO during gestation and lactation. The serum levels of reactive oxygen species (ROS) (*P* < 0.05), 8-hydroxy-deoxyguanosine (8-OHdG) (*P* < 0.05), and thiobarbituric acid reactive substances (TBARS) (*P* < 0.05) were higher during gestation (days 90 and 109) and lactation (days 1 and 3) than in early gestation (day 10). Compared with the control group, the OEO diet significantly reduced sows' serum concentrations of 8-OHdG (*P* < 0.05) and TBARS (*P* < 0.01) on day 1 of lactation. The OEO diet increased the sows' counts of faecal *lactobacillus* (*P* < 0.001) while reducing* Escherichia coli* (*P* < 0.001) and* Enterococcus* (*P* < 0.001). In the third week of lactation the treatment tended to increase sow's feed intake (*P* = 0.07), which resulted in higher average daily gain (*P* < 0.01) of piglets. Our results demonstrated that there is an increased systemic oxidative stress during late gestation and early lactation of sows. The OEO supplementation to sows' diet improved performance of their piglets, which may be attributed to the reduced oxidative stress.

## 1. Introduction

Oxidative stress results from increased production of reactive oxygen species (ROS) or a decrease in antioxidant defense. Oxidative damage is a strong indicator of health status and wellbeing of animals [1]. A recent study showed that pregnant sows had elevated oxidative stress during late gestation and lactation [2], which was responsible for impaired milk production, reproductive performance, and finally longevity of sows [3–5]. Accumulated evidence suggests that excessive ROS affect the insulin signaling cascade, which leads to insulin resistance [6, 7]. Insulin resistance during peripartal period was shown to have a negative effect on lactation feed intake of sows [8, 9]. Thus, dietary antioxidant concentrations need to be reevaluated for their sufficiency in sow diets especially to prevent excessive oxidative stress during gestation and lactation.

Oregano essential oil (OEO) is isolated from plants (*Origanum vulgare* L.) by steam distillation. Chemical analyses of these oils have shown the principal nutraceutical constituents to be carvacrol and thymol [10].* In vitro*, OEO has been reported to possess antimicrobial [11, 12] and antioxidant activities [13, 14]. Although previous studies have reported that dietary supplementation of OEO to sows reduced the fat percentage in milk, did not affect growth pattern of suckling pigs, and increased reproductive performance of sows [15–17], its effect on sows' oxidative stress status during gestation and lactation remains unknown. Therefore, the objective of this study was to examine the effects of dietary supplementation of sow diets with OEO during gestation and lactation on oxidative stress status, colostrum and milk composition, lactation feed intake, and piglet performance.

## 2. Materials and Methods

All experimental procedures were approved by the Animal Care and Use Committee of Huazhong Agricultural University.

### 2.1. Animals, Diet Treatments, and Management

Sixty large white sows were originally allotted to the study; the sows were divided into two groups (control group and OEO group) of 30 animals. After breeding, six sows (4 in control group and 2 in OEO group) were returned to estrus within the estrus cycle. Fifty-four large white sows and parities of 4.95 ± 1.12 were used in this trial. After breeding, the sows were randomly allotted to 1 of 2 experimental dietary treatments based on parity and body weight (BW): control sows were fed a basal diet (Table 1) with no supplementation (C, *n* = 26) and the treatment sows were fed a basal diet added with 15 mg/kg OEO through gestation and lactation (OEO, *n* = 28). The commercially available OEO is a mixture powder that contains 5% OEO of* Origanum vulgare* subsp.* hirtum* plants and 95% natural feed grade inert carrier. For each kilogram of sow feed we supplemented 300 mg Orego-Stim (Meriden Animal Health Ltd, UK), that is, 15 mg OEO. The components of OEO were shown in Table S1 in Supplementary Material available online at http://dx.doi.org/10.1155/2015/525218. The OEO supplement contained carvacrol (81.92%) and thymol (3.50%). Sows from the two groups were restrict-fed with their respective diets during gestation. Sows were fed 2.0 kg/day from days 1 to 30 of gestation, 2.5 kg/day from days 31 to 90 of gestation, and 3.0 kg/day from day 91 of gestation to farrowing. The diets were supplied twice a day (07:00 and 14:30). During lactation, the diet was supplied three times a day (07:00, 11:00, and 17:30) to ensure sows* ad libitum* access to feed. Pregnant sows were housed individually in gestation stalls (2.2 m × 0.7 m × 1.1 m). Sows were moved from the gestation stalls to the farrowing rooms on day 107 ± 2 of gestation and then kept in individual farrowing crates with stalls (2.2 m × 0.7 m) in pens that provided space on both sides of the stall (2.2 m × 0.5 m) for the pigs after birth. Both sows and piglets had free access to water. Piglets were not offered creep feed. Sow milk was the only feed available to the piglets during lactation. During the experimental period, data from sows with illness, serious lameness, death, and reproductive failure were not included in the analyses (Table 2).

### 2.2. Performance Measurement

BW and backfat thickness of sows were measured on days 0 and 107 of pregnancy, within 24 h of farrowing and at weaning. Backfat thickness at 65 mm on each side of the dorsal midline at the last rib (*P*_2_) was measured using ultrasound (PIGLOG105, SFAK- Technology). At farrowing, the numbers of total piglets born and piglets born alive were recorded. The piglets were cross-fostered within dietary treatment groups by 48 h after farrowing to adjust the litter size. The number of piglets per sow ranged from 9 to 12 piglets. At weaning, the numbers of weaned piglets were recorded. Piglets were weighed within 24 h of birth (day 1) and on days 7, 14, and 21. The daily feed intake of sows during lactation was recorded each morning by weighing daily feed refusals.

### 2.3. Samples Collection

At 2 h after feeding on days 10, 60, 90, and 109 of gestation and on days 1, 3, 7, and 21 of lactation, before feeding on days 10 and 109 of gestation and on days 3 and 7 of lactation, blood samples were collected from sows (5 sows per diet group with the similar parity) by ear vein with a minimum amount of stress into heparinized tubes (5 mL) or in tubes containing no anticoagulant (5 mL). Fasting sows were selected for blood sampling after an overnight fasting period of 16 h during gestation and 12 h during lactation. Samples collected for plasma assays (heparinized tubes) were kept on ice and centrifuged for 5 min at 8500 ×g at 4°C. Samples for serum assays (tubes containing no anticoagulant) were left at room temperature for 4 h and then centrifuged for 5 min at 5000 ×g at 4°C. Serum and plasma samples were stored at −80°C until they were assayed. Colostrum samples (30 mL) were collected from the third, fourth, and fifth pairs of mammary glands of sows (5 sows per diet group with the similar parity) within 4 h after the initiation of farrowing. Milk samples (30 mL) were also collected from the third, fourth, and fifth pairs of mammary glands of sows (5 sows per diet group with the similar parity) on day 18 after an intramuscular injection of 10 IU oxytocin behind an ear. The colostrum and milk samples were immediately frozen at −20°C until analysis. Fresh faecal samples were collected from the sows (5 sows per diet group with the similar parity) on day 109 of gestation into individual plastic containers and kept frozen at −20°C.

### 2.4. Quantification of Faecal Bacteria

Bacterial DNA was extracted and purified from faeces samples using a QIAamp DNA stool kit (Qiagen, Germany) in accordance with the manufacturer's instructions. Genomic DNA from faeces was pooled and amplified through routine PCR using species and genus specific primers (Table 4). After PCR amplification with a Taq DNA polymerase kit (Promega, USA) and electrophoresis on a 1.5% agarose gel, PCR products were purified according to the manufacturer's protocol (Omega, USA). The purified PCR products were linked to the pMD18-Tvector system (Takara Bio Inc) and then transferred to* Escherichia coli* DH5*α* (Qiagen, Germany) to clone. After checking the size of the cloned inserts with PCR amplification, the extracted plasmids of the positive clones were sequenced commercially, obtaining the positive plasmids.

Serial dilutions of these positive plasmids served to generate standard curves using quantitative real time PCR (BIO-RAD System, USA), permitting estimations of absolute quantification based on respective gene copies. After 10-fold dilution, microbial genomic DNA was performed to estimate absolute quantification. The reaction was performed in a total volume of 20 *μ*L containing 4 *μ*L template DNA, 1 *μ*L forward and reverse primers, 10 *μ*L iTaq SYBR Green PCR Master Mix (BIO-RAD, USA), and 5 *μ*L nuclease-free water. The thermal cycling conditions involved an initial denaturation step at 95°C for 4 min followed by forty cycles of 95°C for 10 s, annealing temperature (Table 3) for 10 s, and 72°C for 30 s, followed by a product melting curve to confirm the specificity of amplification. The mean threshold cycle values from the triplicate of each sample were used for calculations. The data was calculated as gene copy numbers per gram of wet faeces and presented as Log_10_ CFU/g faeces for the convenience of data analysis.

### 2.5. Analysis of Oxidative Stress Parameters

Serum samples were used to measure levels of thiobarbituric acid reactive substances (TBARS), 8-hydroxy-deoxyguanosine (8-OHdG), glutathione peroxidase (GSH-Px), and reactive oxygen species (ROS). An uncontrolled increase in ROS production leads to peroxidative damage of macromolecules, which, in turn, may cause disturbances in the metabolism and physiology [18]. TBARS is one of the most frequently used indicators of lipid peroxidation and was determined in the current study. The major marker for oxidative damage to nucleic acids, 8-OHdG, was chosen to determine the DNA damage in the current study [19]. Serum samples were analyzed for activities of antioxidant enzymes including GSH-Px and for TBARS using the commercial kits provided by Nanjing Jiancheng Bioengineering Institute (Nanjing, China) [20]. GSH-Px activity was determined based on quantifying the rate of oxidation of GSH to GSSG by H_2_O_2_ catalyzed by GSH-Px. GSH reacts with 5,5′-dithiobis-p-nitrobenzoic acid (DTNB) to produce yellow colored 5-thio-2-nitrobenzoic acid (TNB) that can be quantified spectrophotometrically at 412 nm. TBARS was analyzed based on the reaction with 2-thiobarbituric acid. The resulting pink product was measured spectrophotometrically at 535 nm. An ELISA kit (Dobio Biotech Co., LTD, Shanghai, China) that utilizes an anti-8-OHdG monoclonal antibody to recognize 8-OHdG was used to determine the concentration of 8-OHdG in the serum sample according to the method described by Pialoux et al. [21]. Levels of ROS were measured in serum by chemiluminescence assay using luminol (5-amino-2,3-dihydro-1,4-phthalazinedione, Sigma) as probe. The measurements according to procedure were described in detail by Du et al. [22].

### 2.6. Laboratory Analyses

Crude protein was determined according to AOAC (1990). The milk composition was determined with a near infrared reflectance spectroscopy method by Milk-Scan 134A/B. Immunoglobulin concentrations were assessed in serum (IgG and IgM) and mammary (IgG and IgM) secretions by ELISA using pig polyclonal immunoglobulin-specific kits (Bethyl, Montgomery, USA). Prior to analysis, colostrum and milk were delipidated by centrifugation at 3000 ×g at 4°C for 20 min. Plasma concentrations of glucose and insulin were determined according to the glucose dehydrogenase activity colorimetric assay kit (BioVision Inc., CA, USA) and insulin ELISA kit (Biosource Inc., Sunnyvale, CA, USA) according to the manufacturer's instructions, respectively. All samples were analyzed in duplicate. The indirect methods were used to evaluate insulin sensitivity by homeostasis model assessment (HOMA); HOMA-IR (insulin resistance) = [(fasting insulin, mIU/L)] × (fasting glucose, mmol/L)]/22.5; HOMA-IS (insulin sensitivity) = 1/[(fasting insulin, mIU/L)] × (fasting glucose, mmol/L)] [23].

### 2.7. Statistical Analyses

An individual sow was considered the experimental unit in all statistical analyses. Results were analyzed by ANOVA using the general linear model procedure (SAS 8.0, Inst. Inc., Cary, NC). For sows and litter performances, the model included the effects of treatment and replicate and their interaction. The number of total piglets born was used as a covariate in the analysis of piglet birth weight and total litter weight at birth. The piglet weight and litter weight on day 21 of lactation were subjected to analysis of covariance with the piglet weight and litter weight after cross-foster as the covariate. Variations of oxidative stress parameters and HOMA values were analyzed by ANOVA using the procedure for repeated measurements of SAS. The model included the effects of treatment, physiological stage, and replicate. When an interaction was significant, this was specified in the text. Data were given as means and SEM. Differences between treatment means were significant at *P* < 0.05 and trends identified when *P* > 0.05 but <0.10.

## 3. Results

### 3.1. Oxidative Stress Parameters, Faecal Microbial Counts, and HOMA Values of Sows

**Figure 1:**
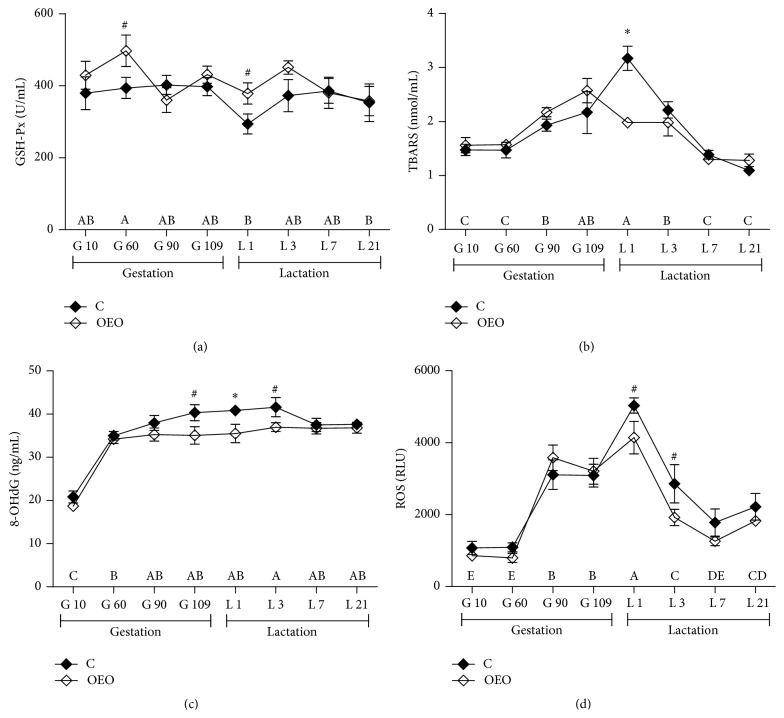
Diet effects on serum levels of GSH-Px (a), TBARS (b), 8-OHdG (c), and ROS (d) of sows (means ± SEM, *n* = 5). ^A–E^Effect of sampling day (*P* < 0.05). ^#^Effect of dietary treatment (*P* < 0.1). ^*∗*^Effect of dietary treatment (*P* < 0.05). There was a treatment × sampling day interaction for serum TBARS concentrations (*P* < 0.05). C = control diet; OEO = 15 mg/kg oregano essential oils diet. GSH-Px, glutathione peroxidase; TBARS, thiobarbituric acid reactive substances; ROS, reactive oxygen species; 8-OHdG, 8-hydroxy-deoxyguanosine; RLU, relative light units.

**Figure 2:**
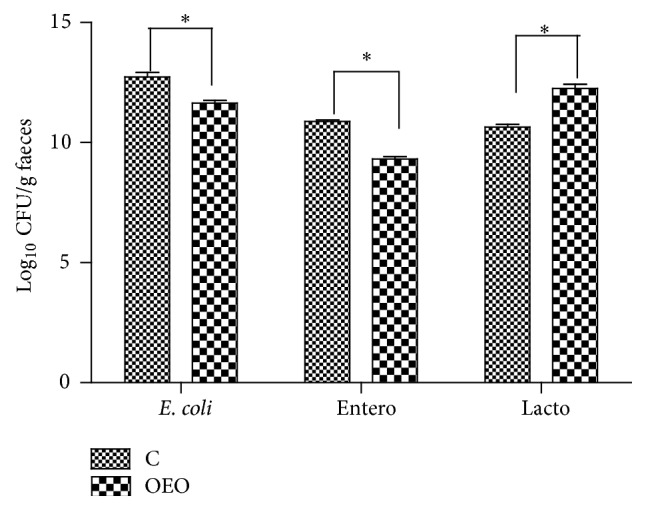
Diet effects on faecal bacterial counts (Log_10_cfu/g) on day 109 gestation of sows. Values are means ± SEM (*n* = 5). ^*∗*^Significant difference between groups, *P* < 0.001. C = control diet; OEO = 15 mg/kg oregano essential oils diet.* E. coli*,* Escherichia coli*; Entero,* Enterococcus*; Lacto,* Lactobacillus*; CFU, colony forming unit.

**Figure 3:**
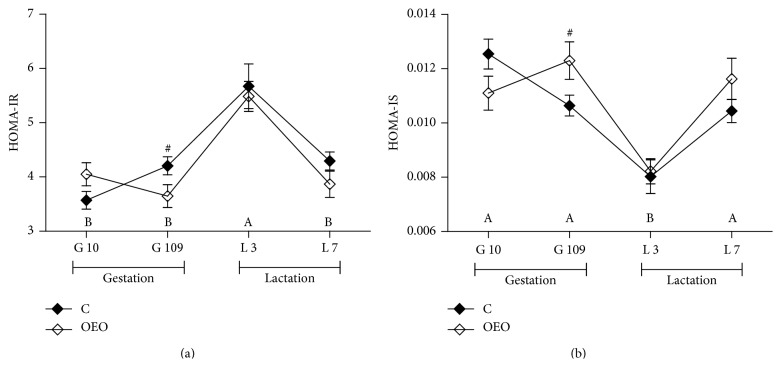
Diet effects on the value of HOMA-IR (a) and HOMA-IS (b) of sows. Plasma glucose and insulin concentrations before feeding were measured, the indirect methods to evaluate insulin sensitivity by homeostasis model assessment (HOMA); HOMA-IR = [(fasting insulin, mIU/L) × (fasting glucose, mmol/L)]/22.5; HOMA-IS = 1/[(fasting insulin, mIU/L) × (fasting glucose, mmol/L)]. Values are means ± SEM (*n* = 5). ^A,B^Effect of sampling day (*P* < 0.01). ^#^Effect of dietary treatment (*P* < 0.1). ^*∗*^Effect of dietary treatment (*P* < 0.05). There was a treatment × sampling day interaction for HOMA-IS value of sows (*P* < 0.05). C = control diet; OEO = 15 mg/kg oregano essential oils diet.

Serum levels of GSH-Px, TBARS, 8-OHdG, and ROS on different days of gestation and lactation are shown in Figure 1. There was a treatment × sampling day interaction for serum TBARS concentrations (*P* < 0.05). The results showed that in both groups serum levels of ROS and TBARS were higher (*P* < 0.05) during late gestation (days 90 and 109) and lactation (days 1 and 3) than in early gestation (day 10). Additionally, in both groups serum concentrations of 8-OHdG were higher (*P* < 0.05) during gestation (days 60, 90, and 109) and lactation (days 1, 3, 7, and 21) than in early gestation (day 10). Compared with the C group, sows under OEO treatment had significantly lower serum concentrations of TBARS (*P* < 0.01) and 8-OHdG (*P* < 0.05) on day 1 of lactation. They tended to have higher serum concentrations of GSH-Px on day 60 of gestation (*P* = 0.08) and day 1 (*P* = 0.07) of lactation, lower serum concentrations of 8-OHdG on day 109 of gestation (*P* = 0.09) and day 3 of lactation (*P* = 0.09), and also lower serum levels of ROS on day 1 (*P* = 0.09) and day 3 (*P* = 0.08) of lactation than sows fed C diet. In addition, the OEO diet significantly increased the counts of faecal* Lactobacillus* (*P* < 0.001) whereas it reduced the counts of* Escherichia coli* (*P* < 0.001) and* Enterococcus* (*P* < 0.001) on day 109 of gestation (Figure 2). It was also found that the OEO diet tended to reduce the value of HOMA-IR (*P* = 0.07) but increased the value of HOMA-IS (*P* = 0.06) of the sows on day 109 of gestation (Figure 3).

### 3.2. Colostrum and Milk Composition and IgG and IgM Concentrations in Colostrum and Serum of Sows

Table 4 showed that the dietary treatments had no effect on the colostrum and milk composition, as well as IgG and IgM in serum and colostrum.

### 3.3. Sow Performance

OEO dietary supplementation of sows during gestation and lactation did not affect the BW and backfat gain during gestation, lactation weight, backfat loss, or weaning-to-estrus of sows (Table 5). Sows in the OEO treatment group tended to increase feed intake in the third week of lactation in comparison to the C group (6.46 versus 6.03 kg/day, *P* = 0.07) (Table 5).

### 3.4. Piglet's Performance

The effect of the dietary treatment on piglet performance is shown in Table 6. There were no differences in the numbers of total piglets born, live-born and weaned. However, sows fed the OEO diet significantly increased average piglet weights at birth (1.56 versus 1.44 kg, *P* = 0.04) and on day 21 of lactation (6.94 versus 6.49 kg, *P* = 0.01). Furthermore, average daily gain (ADG) of piglets during the third week (306.51 versus 273.12 g/d, *P* < 0.01) and on days 1–21 of lactation (252.36 versus 233.61 g/d, *P* < 0.01) were significantly increased for sows in the OEO diet group.

## 4. Discussion

The peripartal period, particularly the delivery, is a critical time for maintaining a balance between the production of free radicals and the incompletely developed antioxidative protection of the fetus and the newborn [24]. Lipid peroxidation and antioxidant status are changed during delivery, and these changes affect the fetus by creating oxidative stress [25, 26]. Our study indicated that not only during delivery but also during late gestation and early lactation the sows suffer from increased oxidative stress indicated by their elevated ROS, 8-OHdG, and TBARS levels. These results were similar to a report from Berchieri-Ronchi et al. [2] which showed that there was an increased systemic oxidative stress during gestation and lactation and that the sows were not fully recovered until weaning.

In our study the OEO diet significantly reduced the concentrations of both TBARS and 8-OHdG on day 1 of lactation. One possible explanation is that sows suffer from the greatest oxidative stress then. In the other two parameters (GSH-Px and ROS), positive effects of supplementing OEO were also found. This is in line with the previous study [27] reports in weaned pigs. This positive effect of OEO could probably be attributed to its composition which mainly contained carvacrol (81.92%) and thymol (3.50%) (Table S1), because both carvacrol and thymol have been reported to scavenge superoxide radicals and hydrogen peroxide [28, 29].

Moreover, the OEO diet increased the counts of sows' faecal* Lactobacillus* but decreased the counts of* Enterococcus *and* Escherichia coli*. Actually, it has been demonstrated that dietary supplementation with essential oils containing carvacrol and thymol decreases populations of* Escherichia coli* in broiler chickens [30] and increases the proportions of* Lactobacillus* in cecum of broilers [31].* Lactobacillus *has the ability to inhibit ROS production through fermentation of colon digesta and to inhibit the growth of* Enterococcus faecalis* and* Escherichia coli* [32]. The results of the present study indicated that sows fed the OEO diet shifted microbial ecology in favor of reducing ROS production that alleviated oxidative stress and oxidative damage of sows. The period of transition between late pregnancy and lactation represents an enormous metabolic challenge to the high-yielding sow. Alleviating oxidative stress could definitively benefit sow's health status.

We also found that supplementing the sow diet with OEO tended to increase lactation feed intake of sows. This observation is consistent with the work of Allan and Bilkei [16]. During pregnancy and lactation, the sow undergoes numerous physiologic and metabolic changes such as progressive and reversible insulin resistance corresponding to a decreased effectiveness of insulin to regulate blood glucose [33]. Moreover, insulin resistance during the peripartal period has negatively impacted the lactation feed intake of sows [8, 9]. Sows fed the OEO diet tended to improve their insulin sensitivity during late gestation (HOMA values, Figure 3). Excessive ROS has been shown to affect the insulin signaling cascade, and then the most common outcome of disrupted insulin signaling is insulin resistance [7]. Thus, we speculated that supplementation of OEO in sow diets may improve insulin sensitivity during late pregnancy by affecting ROS clearance in serum of sows.

In the present trial, sows fed the OEO diet exhibited significantly increased piglets ADG, which can usually indicate an improvement of the amount and/or quality of colostrum and milk, as they are major determinants of litter performance [34]. With regard to the quality of colostrum and milk, our results showed no differences among the dietary treatments in their nutrient compositions and immunoglobulin concentration, contradicting with previous finding of Ariza-Nieto et al. [17] who reported that OEO administered to lactating sows reduced fat percentage in milk on days 7 and 14. Discrepancies may be due to differences between the duration of treatments (gestation and lactation versus lactation) and the dose (15 mg/kg versus 250 mg/kg).

Since no differences of the quality of colostrum and milk were found, the improvement of piglet performance can only be explained by the increase of their amount. Actually we did find a tendency of increased sows' lactation feed intake with the supplementation diets, which resulted in the production of a higher amount of colostrum and milk [35, 36]. It was unexpected to note that sows fed the OEO diet showed more backfat thickness loss during lactation despite their increased lactation feed intake. This might be due to their higher litter weight, which might have pushed them to use their body reserves for milk production.

## 5. Conclusion

Our results demonstrated that there is an increased systemic oxidative stress during late gestation and early lactation of sows. The OEO supplementation to sows' diet during gestation and lactation improved performance of their piglets, which may be attributed to the reduced oxidative stress.

## Supplementary Material

Components of the oregano essential oil.

## Figures and Tables

**Table 1:** Composition of the gestation and lactation diets (as-fed basis).

Item	Gestation	Lactation
Ingredient, %		
Corn	56.30	54.40
Soybean meal, 43% CP	10.00	26.00
Wheat bran	30.40	11.00
Calcium carbonate	1.20	1.51
Dicalcium phosphate	1.04	1.23
Salt	0.40	0.26
Mildewcide^1^	0.12	0.10
Choline chloride	0.14	1.00
Premix^2^	0.40	1.50
Nutrient composition		
Net energy, MJ/kg^3^	9.32	10.36
Crude protein, %	14.06	18.92
Lysine, %^3^	0.61	1.03
Calcium, %^3^	0.77	1.06
Available phosphorus, %^3^	0.33	0.45

^1^Mildewcide: ammonium propionate.

^2^Provided per kg of diet: Cu 30 mg; Fe 160 mg; Zn 160 mg; Mn 55 mg; I 0.5 mg; Se 0.5; Co 0.8 mg; Cr 0.2 mg; Vitamin A 14000 IU; Vitamin D_3_ 2900 IU; Vitamin E 120 mg; Vitamin K_3_ 6 mg; Vitamin B_1_ 2.4 mg; Vitamin B_2_ 8.5 mg; Vitamin B_6_ 4.5 mg; Vitamin B_12_ 0.03 mg; Vitamin H 0.55 mg; Pantothenic acid 30 mg; Folic acid 5 mg; Nicotinamide 50 mg.

^3^Calculated chemical concentrations using values for feed ingredients from the National Research Council (1998).

**Table 2:** The number of sows during the experimental periods.

Item	C^1^	OEO^1^
Breeding	26	28
Culled during gestation^2^	3	1
Parturition	23	27
Culled during lactation^2^	1	2
Weaning	22	25

^1^Dietary treatments: C = control diet; OEO = 15 mg/kg oregano essential oils diet.

^2^Data of sows that were ill, seriously lame, died during the study and had reproductive failure were not included.

**Table 3:** Species and genus specific primers used for real time PCR to profile selected bacteria.

Target group	Sequence of primers (5′-3′)	Product size (bp)	Annealing temperature (°C)
*Escherichia coli*	CATGCCGCGTGTATGAAGAA	96	60
	CGGGTAACGTCAATGAGCAAA		

*Enterococcus*	CCCTTATTGTTAGTTGCCATCATT	144	61
	ACTCGTTGTACTTCCCATTGT		

*Lactobacillus*	AGCAGTAGGGAATCTTCCA	341	58
	CACCGCTACACATGGAG		

**Table 4:** Effects of dietary supplementation of oregano essential oils to sows in gestation and lactation on the colostrum and milk composition and IgG and IgM concentrations in colostrum and serum of sows.

Item	C^1^	OEO^1^	SEM	*P* value
Number of sows	5	5		
Colostrum^2^				
Fat (%)	5.78	4.60	0.36	0.14
Lactose (%)	1.98	2.01	0.07	0.84
Protein (%)	15.02	15.88	0.60	0.51
Solid not fat (%)	20.50	21.34	0.53	0.47
Total solids (%)	27.10	26.58	0.73	0.75
IgG (mg/mL)	15.80	15.79	0.46	0.91
IgM (mg/mL)	2.86	2.85	0.14	0.97
Milk (%),^3^ d 18 of lactation				
Fat	8.71	8.10	0.56	0.61
Lactose	4.84	4.79	0.12	0.85
Protein	4.56	4.60	0.06	0.77
Solid not fat	13.60	13.59	0.11	0.97
Total solids	22.18	21.64	0.40	0.53
Serum, d 109 of gestation				
IgG (mg/mL)	8.90	7.82	0.46	0.27
IgM (mg/mL)	2.69	2.82	0.15	0.69

SEM, standard error of means.

^1^Dietary treatments: C = control diet; OEO = 15 mg/kg oregano essential oils diet.

^2^Colostrum was collected within 4 h after the initiation of farrowing.

^3^Sows were injected with 10 IU of oxytocin intramuscularly behind the ear to induce milk ejection.

**Table 5:** Effects of dietary supplementation of oregano essential oils to sows in gestation and lactation on sow performance.

Item	C^1^	OEO^1^	SEM	*P* value
Number of sows	22	25		
Daily allowances during gestation, kg/d	2.42	2.42	0.01	0.99
Sow BW, kg				
Breeding	232.7	234.0	4.01	0.59
Gestation, day 107	267.3	268.1	3.31	0.88
Gain	34.6	34.1	2.08	0.33
Parturition	246.3	247.3	3.30	0.98
Weaning	236.3	237.0	3.40	0.75
Loss	10.0	10.3	1.54	0.45
Sow backfat thickness, mm				
Breeding	16.2	16.0	0.43	0.78
Gestation, day 107	17.0	17.5	0.47	0.88
Gain	0.8	1.6	0.35	0.79
Parturition	16.3	17.0	0.49	0.46
Weaning	14.4	14.1	0.37	0.56
Loss	1.9	2.9	0.34	0.46
Average daily feed intake, kg				
1st week of lactation	4.21	4.17	0.15	0.88
2nd week of lactation	5.93	5.90	0.15	0.88
3rd week of lactation	6.03	6.46	0.17	0.07
Mean of 1st week to 3rd week	5.39	5.51	0.13	0.55
WEI, d	4.89	4.63	0.11	0.21

SEM, standard error of means; BW, body weight; WEI, weaning-to-estrus interval.

^1^Dietary treatments: C = control diet; OEO = 15 mg/kg oregano essential oils diet.

**Table 6:** Effects of dietary supplementation of oregano essential oils to sows in gestation and lactation on piglet performance.

Item	C^1^	OEO^1^	SEM	*P* value
Number of sows	22	25		
Litter size, number/litter				
Total born	11.59	11.28	0.51	0.65
Born alive	11.41	11.16	0.52	0.71
After cross-foster	10.00	9.76	0.17	0.50
Pigs weaned	9.45	9.60	0.18	0.70
Litter weight, kg				
At birth	16.29	17.24	0.68	0.36
After cross-foster	15.63	15.94	0.41	0.70
At day 7	26.99	27.77	0.72	0.59
At day 14	43.77	46.06	1.00	0.26
At day 21	61.17	66.51	1.42	0.06
Piglet mean BW, kg				
At birth	1.44	1.56	0.09	0.04
After cross-foster	1.57	1.63	0.03	0.30
At day 7	2.70	2.84	0.05	0.18
At day 14	4.57	4.78	0.07	0.12
At day 21	6.49	6.94	0.09	0.01
Piglet ADG, g/d				
Week 1	161.41	172.53	4.14	0.18
Week 2	264.12	276.53	4.10	0.13
Week 3	273.12	306.51	5.90	<0.01
Days 1–21	233.61	252.36	3.51	<0.01

SEM, standard error of means; BW, body weight; ADG, average daily gain.

^1^Dietary treatments: C = control diet; OEO = 15 mg/kg oregano essential oils diet.
