# Supplementary material for: Effects of Dietary Supplementation of Oregano Essential Oil to Sows on Oxidative Stress Status, Lactation Feed Intake of Sows, and Piglet Performance
Source: Biomed Res Int. 2015 Oct 11;2015:525218. doi: 10.1155/2015/525218 (PMC4619846; doi:10.1155/2015/525218)
Supplement: Supplementary file 1 — Components of the oregano essential oil. [file 525218.f1.pdf]

Supplementary Information

MATERIALS AND METHODS

Chemicals

The composition of OEO were analyzed in the laboratory using Hewlett-Packard 6890 gas chromatograph equipped with a cross-linked 5% PH ME siloxane Hewlett-Packard-5MS capillary column (25m × 0.25mm ID, 0.25 μ m film thickness), coupled to a Hewlett-Packard 5972A mass spectrometer (Hewlett Packard Ltd., Bracknell, UK). Relative percentage amounts were obtained directly from GC peak areas. The absolute concentration of carvacrol was determined by gas chromatography using external standard method. The GC operating conditions were the same as above. The absolute concentrations of other ingredients were calculated based on the relative percentage amounts (Table S1).

Table S1. Components of the oregano essential oil

Chemical constituents	Percentage	Absolute concentration (mg/ml)
α -Thujene/ α -pinene	0.66	0.29
Camphene	0.09	0.04
β -Pinene	0.07	0.03
Sabinene	0.04	0.02

Myrcene	0.86	0.38
α -Phellandrene	0.08	0.04
α -Terpinene	0.58	0.25
Limonene	0.13	0.06
1,8-Cineole+ β -phellandrene	0.09	0.04
β -Ocimene	0.07	0.03
γ -Terpinene	4.49	1.97
3-Octanone	0.07	0.03
p-Cymene	3.07	1.35
Terpinolene	0.04	0.02
3-Octanel	0.01	＜0.01
1-Octen-3-ol	0.24	0.11
Dimethyl styrene	0.01	0.00
Trans-sabinene hydrate	0.10	0.04
Linalool	0.28	0.12
Cis-sabinene hydrate	0.06	0.03
1-Terpilool	0.04	0.02
Terpine-4-ol	0.34	0.15
Carvacrol methyl ether	0.22	0.10
β -Caryophyllene	1.41	0.62
Dihydrocarvone	0.08	0.04
α -Humulene	0.14	0.06
α -Terpineol	0.16	0.07
Bomeol	0.30	0.13

β-Bisaholene	0.70	0.31
Caryophyllene oxide	0.14	0.06
Thymol	3.50	1.54
Carvacrol	81.92	36.01
Total	99.99	
